# Characterizing Enterotypes in Human Metagenomics: A Viral Perspective

**DOI:** 10.3389/fmicb.2021.740990

**Published:** 2021-09-29

**Authors:** Li Song, Lu Zhang, Xiaodong Fang

**Affiliations:** ^1^College of Life Sciences, University of Chinese Academy of Sciences, Beijing, China; ^2^Department of Computer Science, Hong Kong Baptist University, Kowloon, Hong Kong SAR, China

**Keywords:** gut metagenomics, virome, enterotype, covariate, perspective

## Abstract

The diversity and high genomic mutation rates of viral species hinder our understanding of viruses and their contributions to human health. Viral enterotypes as a description of the gut virome, its characteristics have not been thoroughly studied. Here we investigated the human gut virome composition using previously published sequencing data of 2,690 metagenomes from seven countries with various phenotypes. We found that the virome was dominated by double-stranded DNA viruses in our data, and young children and adults showed different stages in their fecal enterovirus composition. Beta diversity showed there were significantly less homogeneous in individuals with severe disorders of bile acid secretion, such as cirrhosis. In contrast, there were no significant differences in distances to centroids or viral components between patients with phenotypes unrelated to bile acid, such as hypertension. Enterotypes determined independently from various projects showed similar specific viruses and enrichment direction. Confounding factors, such as different sequencing platforms and library construction, did not confuse enterotyping. The gut virome composition pattern could be described by two viral enterotypes, which supported a discrete, rather than a gradient, distribution. Three main components, enterotype 1 and 2 specific viruses and the other, comprise the total viral variation in these sets. Compared with enterotype 2, enterotype 1 had a higher viral count, Shannon index, and similarity between samples. The relative abundance of enterotype-specific viruses is a crucial determinant of enterotype assignment. Samples not matching any of the defined enterotypes in the database did not necessarily correlate to sickness. Therefore, the background context must be carefully considered when using a viral enterotype as a feature for disease prediction. Our results highlight important insights into the human gut virome composition by exploring two-main viral enterotypes in population and providing an alternate covariate for early disease screening.

## Background

In recent years, many studies have shown that viral colonization in the human body is highly related to human health and life. Cross-species virus transmission poses a potential threat to human and animal health (Daszak et al., 2000). With advanced sequencing technology, viral genomes (virome) have become a new material for viral research, which enables viral identification and classification at the molecular level (Fujimoto et al., 2020; Gregory et al., 2020). The success of virome studies greatly relies on high-quality viral genomes (Minot et al., 2011). Viruses are highly diverse and individual-specific (Reddy et al., 2015) and traditional purification strategies, culture, and sequencing are robust in other scenes. Still, they are not suitable to build a virus genome database (Paez-Espino et al., 2016), thus severely preventing comprehensive and intensive studies for human gut virome.

The strategy of assembling the viral genome involves a comprehensive and in-depth analysis of the virome. Paez-Espino et al. (2016, 2017, 2019) and Roux et al. (2021) launched the “Uncovering Earth’s virome” project to mine viral sequences ignored within metagenomic data and built the Integrated Microbial Genome/Virus (IMG/VR) database in 2016. Recently, 28,060 metagenomes were used to mine 142,809 human gut viruses, and Gubaphage was found to be the second common virus branch in the human gut (Camarillo-Guerrero et al., 2021). These projects built relatively thorough virus databases much larger than viral RefSeq^1^ and laid the foundation for a comprehensive analysis of the human gut virome (Gregory et al., 2020). The disease is associated with certain viromic compositions in the gut, but most studies have ignored the importance of viral sequencing information in massive metagenome sequencing data. The construction of multiple viral genome databases has enabled detailed research on the human gut virome.

Enterotypes were suggested as predicted clusters of microbes in the human gut, and it describes the distributional of the human gut microbial community (Arumugam et al., 2011). Multiple studies have suggested that there are two dominant enterotypes, which correspond to the individuals’ preference for digesting plant fiber or animal meat (Costea et al., 2017). The gut is an ecosystem, and the enterotype summarizes its microbial characteristics using mathematical methods (Arumugam et al., 2011; Holmes et al., 2012), but such knowledge is insufficient to depict a dynamic ecosystem (Jeffery et al., 2012). Research on the composition patterns and function of the gut microbiome will significantly improve our understanding of its relationship with health and disease (Knights et al., 2014). Enterotypes may be used for gut microbial analysis to inform disease treatment and prevention strategies, and may also provide a theoretical basis for diet therapy. The relationship between viral enterotypes and the human disease status is still largely unknown. Whether enterotypes can be used as biomarkers for predicting the disease status requires further research.

In this study, we collected previously published human metagenomic sequencing data, conducted sample quality control through a fast pipeline, identified viral species, and determined viral abundance. Furthermore, we established a database of the human gut virome based on 2,690 metagenomes. We observed the relationship between viral species and abundance in various ethnicities, countries, and diseases using different DNA library construction methods and sequencing platforms, and analyzed the association between viral community diversity and disease. Viral enterotypes were assigned by the Dirichlet multinomial mixture model (DMM). We independently identified enterotype-specific viral operational taxonomic units (vOTUs) for each dataset and resolved the inter-relationships among enterotypes from different projects by comparing the abundance of enterotype-specific viruses. Further, we compared the ecological diversity of viruses between different enterotypes, and evaluated the correlation of viral enterotype disorders and their diversity with diseases. The results of study elucidate the relationship between dsDNA virome and human health in a large population.

## Materials and Methods

### Choosing an Alignment Method

FastViromeExplorer (Tithi et al., 2018) was used to map all reads to the reference and apply the expectation-maximization algorithm to estimate the viral species and their corresponding abundance. This software uses Kallisto (Bray et al., 2016), which is based on *k-mer* alignment. It introduces three criteria to improve the performance of virus detection. First, by calculating the ratio of the observed to the expected extend of genome coverage, if this ratio is less than 0.3, the virus is considered a false positive and should be discarded. The main goal of this criterion is to remove viruses with only duplicated regions being aligned. Second, by evaluating genome coverage. If less than 10%, remove the sequence. Last, when less than 10 reads could be aligned to the virus genome, the virus is considered a false positive. Ajami et al. (2018) found that FastViromeExplorer has high virus detection sensitivity and specificity and recommended using it.

### Data Collection and Processing

We downloaded all the data from the National Center for Biotechnology Information (NCBI) sequence read archive (SRA). The SRA numbers for each project were listed in Supplementary Table 1. We only chose pair-end data from projects sequenced by the Illumina HiSeq 2000 or 2500 platforms. After preprocessing the original data (Supplementary Figure 1), we used Trimmomatic (Bolger et al., 2014) with default parameters to remove the adapters and low-quality reads. Then, Kneaddata^2^ was used to detect and remove contamination from the host’s DNA (B37 version) and RNA (RefSeq GRCh37.p13 full version) data, and discarded the unpaired reads. Finally, we used FastViromeExplorer to align reads to IMG/VR v2 (IMG_VR_2018-07-01_4—IMG/VR v2) and carried out the taxonomic assignation and calculated their abundance.

### Viral Contig Taxonomic Annotation

We used Glimmer3 toolkit Version 3.02b (Delcher et al., 2007) to predict and extract the open reading frames of viral contigs with a minimum length threshold of 100 amino acids. The predicted protein sequences were aligned to the UniProt TrEMBL database as of February 2021 (Bateman et al., 2021) using BLASTX (Boratyn et al., 2012). The major voting system was then used, as described previously, to ascertain the family of a viral contig (Zuo et al., 2020). A contig needed to be supported by five protein hits. When a virus sequence was annotated to multiple families, we chose the family with the largest number of aligned proteins. When multiple families had the same number of proteins, the size of the accumulated *E*-value (BLASTX alignment) of all proteins was compared.

### Statistical Analysis

We first used Tximport (Soneson et al., 2015) R package to read the original abundance information of the virus (the output of Kallisto) from each project. Function “specaccum” in vegan (version 2.5-7) with parameters “method = “random,” permutations = 100” was used to do rarefaction analysis. For alpha diversity, we used our in-house script to calculate observed richness and Shannon index. Vegan R package was used for calculating beta diversity. “vegdist” function with method = “bray” was used to calculate distance matrix for beta diversity, Principal Coordinates Analysis (PCoA) was done by “pcoa” function with parameters “correction = ‘none,’ rn = NULL.” Adonis with option “permutations = 100,000” was used to analyzing ecological differences among viruses between cases and controls. “betadisper” was used to calculate the distance from the centroid. The Kruskal test was used to determine whether there was a significant difference in distance between cases and controls. The *p*-values were adjusted by R “p.adjust” with option “method = ‘BH’”, and “sumlog” in metap (version 1.4)^3^ was used to calculate meta *p*-value.

### Enterotyping and Maaslin2 Analysis

Data on enterotypes could be used to help adjust population stratification in Metagenome-wide association studies (MWAS) analysis (Wang et al., 2012; Schmidt et al., 2018). The correlation between enterotypes and disease phenotypes had received much attention in this field. The DMM method was commonly used for determining enterotypes of the gut microbiome (Ding and Schloss, 2014). Different library construction methods, sequencing platforms, and other factors might lead to the false-positive assignment of enterotypes. To avoid this situation, we adopted a project-independent strategy for determining enterotypes. Enterotypes were assigned using the “DirichletMultinomial” R package, with predetermined parameters of 1–10 enterotypes, and enterotype data from each project was run 10 times. The smallest Laplace value corresponding to the number of enterotypes was considered as the optimal result. MaAsLin2^4^ analysis was used to determine the specific vOTUs associated with enterotypes, with correlations considered significant at the 5% level (after multiple testing correction). We applied the “envfit” function in vegan to estimate the effect size of the structural variance explained by factors such as enterotype and disease.

### Manual Categorizing Enterotypes and Random Pairing Permutation

We focused on projects with 2–3 enterotypes. Enterotype-specific vOTUs were determined independently using MaAsLin2 in each project, and then we summarized the vOTUs shared ratio among projects. The results showed high consistency. Then, we grouped the samples of each project by enterotype. We calculated the average relative abundance of vOTUs for each group, and built the relationship between vOTUs and enterotypes by defining group-specific vOTUs with the greatest relative abundance. Further, we found that some groups had the same vOTUs in different projects. By this apparent group similarity, we manually clustered all groups, and formed three enterotypes. To prove the validity of this classification strategy, we involved randomly paired enterotypes from different projects. We assumed that paired enterotypes had the same specific vOTUs and enrichment directions. We assigned a lower error rate to paired enterotypes if they had more identical vOTUs and similar enrichment trends. We repeated pairing 5 million times to obtain the distribution of pairing scores.

## Results

### Sequencing Data and Summarization

We collected 12.36 TB of metagenomic sequencing data from 18 previously published projects (Supplementary Tables 1, 2). We selected data from 2,690 metagenome samples of high quality for the subsequent analysis (Supplementary Figure 1 and Supplementary Table 1), of which 1,092 samples were from women, 859 were from men, and 739 were from unknown sex. The length of sequencing reads from each sample were 2.26–8.55 G (Supplementary Table 1), and approximately 10% of strictly filtered reads were aligned against IMG/VR v2 viral sequences (Supplementary Table 3). We obtained 2,690 metagenome samples by choosing paired-end sequencing data from the Illumina HiSeq 2000 and 2500 platforms and excluding projects with a small data size (<1 G).

**FIGURE 1:**
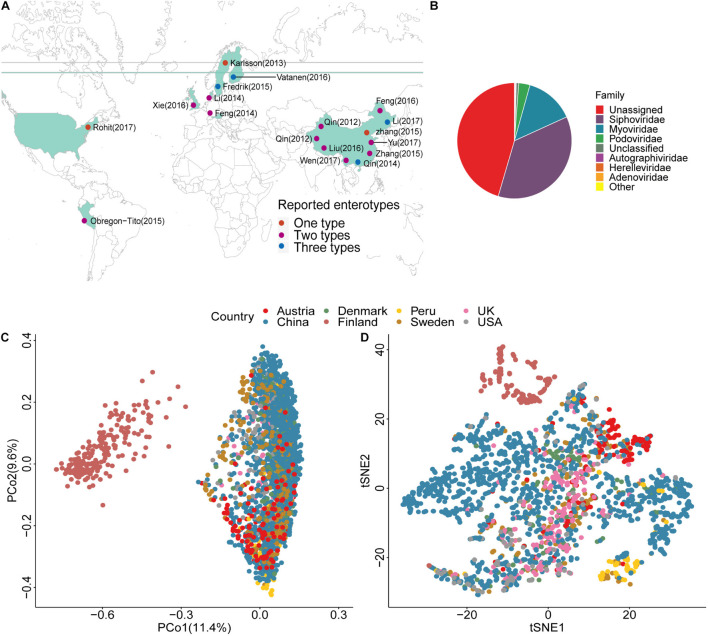
Location, taxonomic assignment, and abundance of the 2,690 samples. **(A)** Geographic locations of the 18 projects, with classification by the number of enterotypes. **(B)** Pie chart shows viral taxonomic assignment at the family level by protein alignment. **(C)** Principal Coordinates Analysis (PCoA) plot based on the Bray–Curtis distance and the relative abundance of viruses. **(D)** t-Distributed Stochastic Neighbor Embedding (tSNE) plot based on the relative abundance of viruses.

We annotated the geographic locations of the included projects on the basis of their predominant samples (Figure 1A). Because there were no specific sampling coordinates, each project was located by country. We annotated the viral taxonomy at the family level based on the protein sequence similarities (Minot et al., 2013; Hannigan et al., 2015). Approximately 50% of the viral genomes failed taxonomic assignment (Figure 1B and Supplementary Table 4), and double-stranded (ds) DNA viruses, such as Siphoviridae, Myoviridae, and Podoviridae, were the dominant enteroviruses as previously reported (Zuo et al., 2020). The density peak was close to zero, which indicated that the viruses were rarely shared among individuals (Supplementary Figure 2). The samples from Finland were outliers in the PCoA and tSNE plots (Figures 1C,D and Supplementary Figure 3) because of the low viral diversity (Supplementary Figure 4). This finding might be explained by age. The average age of individuals in the Finland project was 1.5, and their gut communities did not reach stable states. The first principal coordinate explained 11.4% of the variance, which firstly separated Finland and was most likely due to age. The second and third principal coordinates explained 9.6 and 6.4%, respectively. It was a small proportion of the variance. A possible explanation was that the human gut virome might correlate with many factors (Gregory et al., 2020), and each had a small contribution. Although the samples from the other six countries showed substantial variability in the PCoA and tSNE plots (Figures 1C,D), they belonged to the same cluster, especially the samples from the studies conducted in China. The studies from China had the most individuals, and the samples were spread over almost the entire plot. In the tSNE plot, we found that the samples from Austria and Peru were clustered in a local region, indicating that the gut virome showed geographical distribution characteristics (Gregory et al., 2020).

**FIGURE 2:**
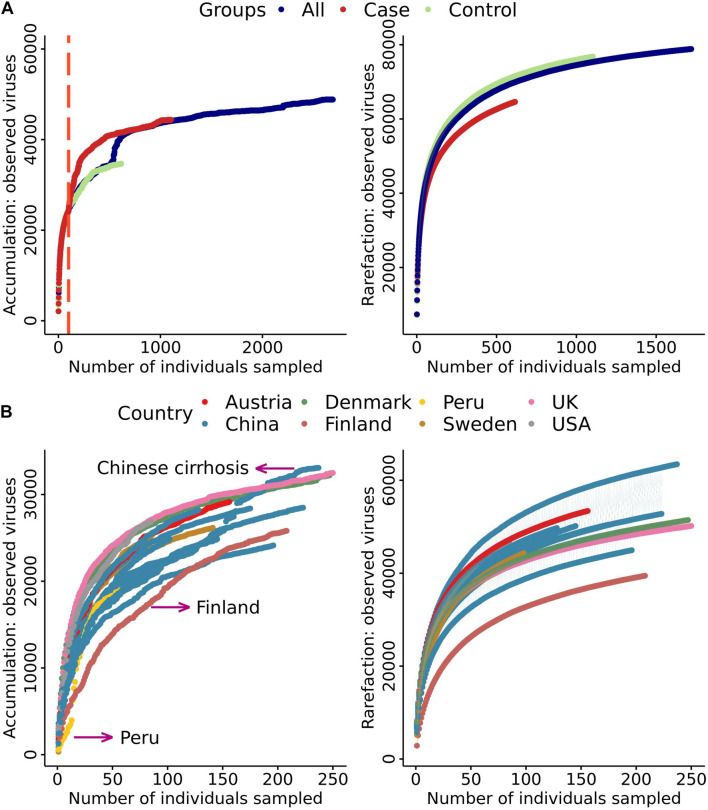
Cumulative curves of the number of virus species against the number of samples. **(A)** Cumulative and rarefaction curves of cases, controls, and all samples. Only samples from studies with a case–control design were included. **(B)** Cumulative and rarefaction curves of sample data divided into seven countries.

To study the distribution characteristics of the viral species in samples with different phenotypes, we divided all samples from studies with a case-control design into three categories. These categories of controls, cases, and all represented healthy people, patients with various diseases, and all individuals, respectively. As more samples were included, the number of viral species showed exponential growth, with no significant difference between cases and controls until samples from ∼100 individuals were included (Figure 2A, left). After including ∼100 individuals, the “case” curve showed a steep increased viral count. As expected, a significant increment in the number of viral species was observed when the number of samples was increased in the “all” curve. However, the three growth curves were essentially parallel (Figure 2A, left), which suggested that the overall number of viruses in the patient population after viral community disruption was limited. More interestingly, the “case” and “all” curves overlapped with each other after ∼1,000 samples. The reason for this finding could be that the case population contained all species of viruses in the control population. In the rarefaction analysis, we randomly disrupted the sample order for 100 permutations. We found that the growth curve of “case” had the highest slope (Figure 2A right), indicating more viral taxa were present in the case group. When we compared the growth curves of different projects, we found that the curves for Finland, Peru, and Chinese populations with cirrhosis had significant differences (Figure 2B left). The samples from the Finland project were obtained from only 1.5-year-old children, at which age the enterovirus community is not well established. It is unclear why the number of viral species in Peru samples was small at the beginning of the curve. The dramatic increase in the number of viruses in the Chinese population with cirrhosis may be due to severe disruption of the enterovirus community. In the rarefaction analysis, rarefaction curve showed Finland had a lower number of virus species. Therefore, we removed Finland in the downstream analysis (Figure 2B right). We used unique species in cases and controls to define group-specific viruses and compared the change in the proportion of unique viral species between cases and controls (Supplementary Table 5). We found that the mean proportion of viruses in case samples was 26% and in control samples was 14%. Among all samples, the proportion of viruses that were unique to cases was 23%. Each case individual had an average of 10.99 viruses to their set, and the percentage of viruses that were unique to controls was 4%, and each control individual had an average of 2.43 unique viruses.

### Relationship of Ecological Diversity of Viruses and Disease

**FIGURE 3:**
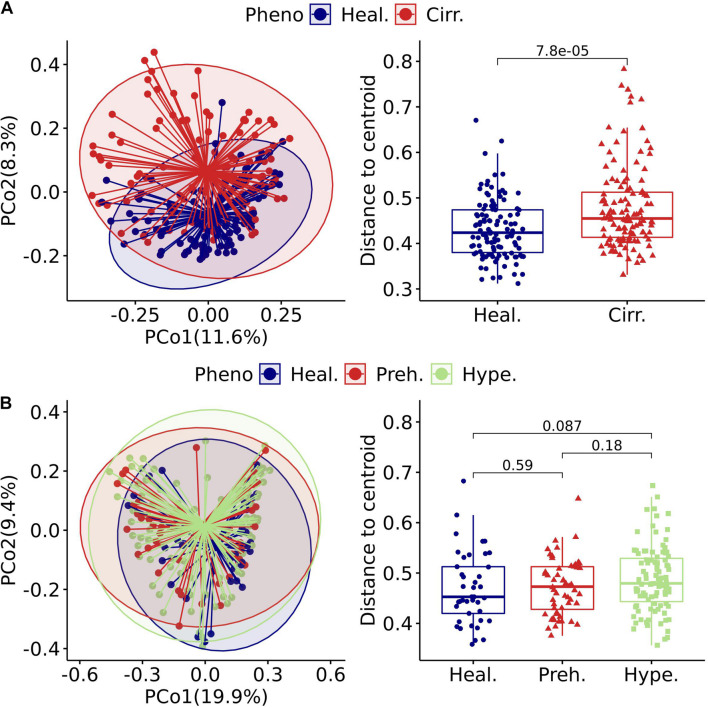
Gut virome characterized by beta diversity in the included projects. **(A)** left: Principal coordinates analysis plot of the cirrhosis project. Each ellipse represents a cohort, and the point connected by the straight gray lines represents the centroid. **(A)** Right: Boxplot of the distance to the centroid. A significant difference in the distance to the centroid was found between the two groups. **(B)** Left: Principal coordinates analysis plot of the hypertension project. **(B)** Right: Boxplot of the hypertension project with comparison for each pair of the three groups. Healthy (Heal.), Cirrhotic (Cirr.), Prehypertension (Preh.), Hypertension (Hype.).

The beta diversity of a microbial community is usually used to evaluate dynamic changes in an ecosystem (Koleff et al., 2003). A comparison of the results of projects with a case-control design revealed that the degree of imbalance in the viral community composition was related to the severity of the disease phenotype. An example of this finding is that the viral community in patients with cirrhosis (Figure 3A left) was significantly different from that in healthy people (Adonis, *p* = 1.00E-04, Table 1). Comparison of the distance to the centroid between patients and healthy individuals by the Mann–Whitney *U*-test showed a significant dissimilarity (Figure 3A right). Specifically, patients had a significantly larger distance than healthy individuals, which indicated that patients might have a considerably disordered viral community. In contrast, we did not detect a significant difference between patients and healthy individuals in the hypertension project (Adonis, *p* = 0.14, Table 1 and Figure 3B left). We also compared the distance to the centroid for each pair of three cohorts, and none of the comparisons were significant (Figure 3B right).

**TABLE 1:** Beta diversity for measuring the sample distance in projects with a case–control design.

Project	R^2^	Adonis for disease	Adjusted *p*-value	Kruskal test	Adjusted *p*-value
Sweden T2D*	1.94%	7.10E-03	1.18E-02	0.37	0.52
China cirrhotic	2.74%	1.00E-05	1.00E-04	7.82E-05	7.82E-04
China rheumatoid arthritis	0.76%	0.08	0.10	0.28	0.47
Austria carcinoma	2.57%	1.90E-04	9.50E-04	0.67	0.84
China colorectal cancer	1.27%	2.14E-02	3.06E-02	0.12	0.40
China hypertension	1.30%	0.12	0.14	0.16	0.40
China coronary heart disease	2.27%	4.10E-04	1.37E-03	0.26	0.47
China T2D discovery	1.27%	5.54E-03	1.11E-02	0.08	0.38
China T2D validation	1.00%	1.79E-03	4.47E-03	0.95	0.95
China obesity	2.62%	0.42	0.42	0.76	0.84

*Type 2 diabetes (T2D).

We further investigated statistical differences in gut viral composition between case and control samples from various aspects to investigate changes in the viral community across different phenotypes. Using Adonis, we found that many projects had significant differences between cases and controls in the human gut viral community (Table 1). Consistently, in cases with relatively mild phenotypes, such as hypertension, rheumatoid arthritis, or obesity, there were no noticeable differences in body metabolism compared with the controls. We found that the cirrhosis cohort showed a substantial difference between the two centroids (Table 1). The Kruskal-Wallis test was performed to determine whether the distance to the centroid in principal coordinates analysis was significantly different between the case and control groups. Compared with the controls, cases with more severe phenotypes, such as cirrhosis and cancer, showed substantial differences in gut viral composition (Table 1 and Supplementary Figure 5), whereas cases with relatively mild phenotypes, such as hypertension, showed no significant differences.

### Characterizing Viral Enterotypes

The characteristics of enterotypes of the gut virome were the focus of this study. There were two or three enterotypes in most projects, while some projects only had one enterotype (Figure 1A, Table 2, Supplementary Figure 6, and Supplementary Table 6). Enterotypes with the same intrinsic composition pattern were considered as the same (Supplementary Figure 7). We used Maaslin2 to discover enterotype-specific vOTUs and then determined their enrichment direction on the basis of mean abundance (Supplementary Table 7). The same enterotype had the same specific vOTUs and the same enrichment directions. We manually classified enterotypes in all of the projects into three groups (Table 2 and Supplementary Table 8). Enterotypes 1 and 2, which are the two major types, were widely distributed in all projects, which indicated that these two types of enterotypes were common across the project populations. However, enterotype 3 was rare. Unclassified individuals were not able to be confidently assigned to enterotype 1 or 2.

**TABLE 2:** Manually categorized results for each project.

Enterotype	Enterotype 1	Enterotype 2	Enterotype 3
Denmark no phenotype	GP1	GP2	−
China cirrhotic	GP2	GP1	GP3
Sweden mother-offspring pair	GP3	GP2	GP1
China rheumatoid arthritis	GP1	GP2	−
Austria carcinoma	GP1	−	GP2
UK no phenotype	GP1	GP2	−
China colorectal cancer	GP1	GP2	−
China hypertension	GP2	GP3	−
China coronary heart disease	GP1	GP2	−
China T2D discovery	GP1	GP2	−
China T2D validation	GP1	GP2	−
China healthy Mongolian	GP1	GP2	−
China ankylosing spondylitis	GP1	GP2	−

*Groups in the same column were considered to belong to one enterotype.*

**FIGURE 4:**
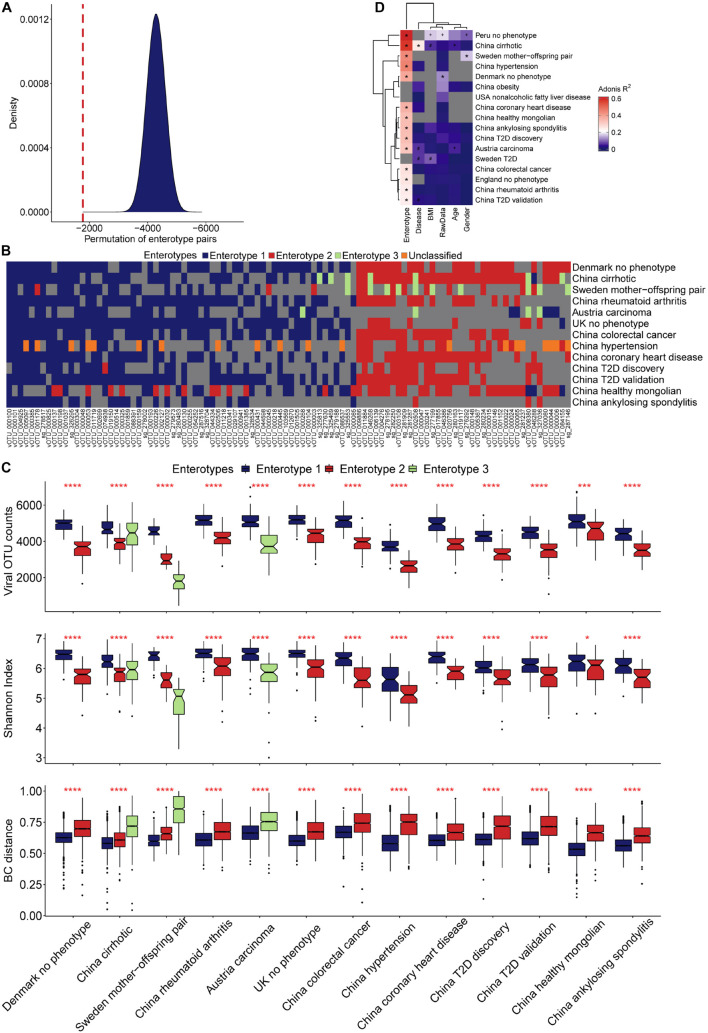
Characterization of viral enterotypes in all projects. **(A)** We used the random pairing method to confirm the accuracy of artificial enterotype classification. The density map shows the score distribution of 5 million permutations, and the red line indicates the score of the manual category. **(B)** The categories of manual enterotypes in different projects show a high concordance of their specific vOTUs and enrichment direction. **(C)** Ecological diversity of different viral enterotype populations. **(D)** Effect of different covariates on the structural variance of the gut virome community.

A permutation test with a scoring strategy was performed to demonstrate the validity of manual classification. These scores showed that our manually classified enterotypes had the lowest error rate (Figure 4A). Moreover, random pairing supported the three major enterotypes. Enterotypes 1- and 2-specific vOTUs were dominant (Figure 4B). The same enterotype-specific vOTUs with highly consistent enrichment trends indicated that the enterotypes from different projects had a similar pattern of virome composition (Figure 4B). Different DNA processing methods, sequencing platforms, ethics, age, and other confounding factors did not affect the identification of viral enterotypes. The vOTUs that were specific to unclassified enterotypes appeared complex. They intersected with either enterotype 1 or 2. Enterotype 3-specific vOTUs in different projects were less concordant than enterotypes 1- and 2-specific vOTUs.

The microbiome is an ecosystem, the stability of which is reflected by the diversity of species in the system. As a species becomes more prosperous and uniform, the system’s diversity increases, and it becomes more resistant to the effects of the external environment (Keesing et al., 2010). Although the viral count varied among samples from different projects, enterotype 1 across the samples had more viruses than enterotype 2 (Figure 4C). A higher value of the Shannon index and a smaller sample distance in enterotype 1, compared with enterotype 2, indicated its more homogeneous composition pattern. We found that more individuals were categorized as enterotype 1 than enterotype 2 (1,204 vs. 716). By comparing the proportion of healthy samples with the two enterotypes, we found that individuals who were categorized as enterotype 2 had a higher risk of being sick than those who were categorized as enterotype 1 (odds ratio: 1.38, Fisher’s exact test, *p* = 0.01), The multi-projects meta-analysis also verified it (Supplementary Table 9). We observed an interesting finding when we compared samples from the cirrhosis project and the Sweden mother-child project. The third enterotype had the most discrete sample distribution in the cirrhosis project, and a higher viral count and Shannon index compared with the Sweden mother-child project (Figure 4C). In contrast, the third enterotype had a large sample distance and the lowest viral count and Shannon Index in the Sweden mother-child project.

**FIGURE 5:**
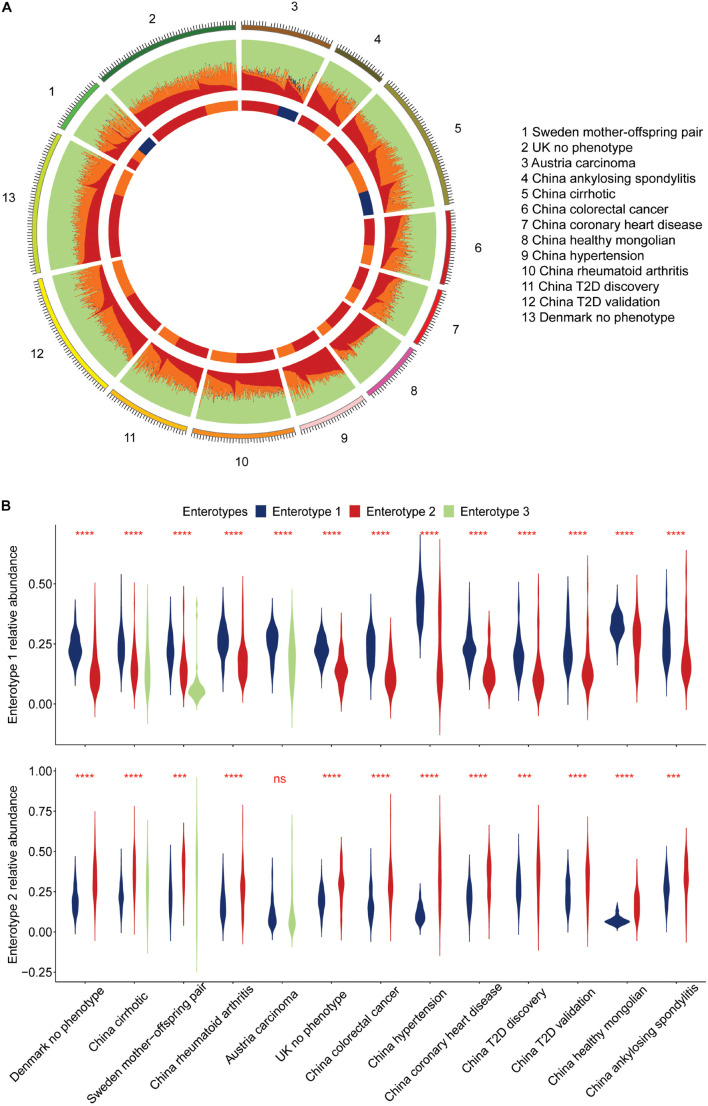
Approximate composition of the human viral community. **(A)** We classified the human viral community into four categories: enterotypes 1, 2, 3 specific viruses, and the others, represented by different colors. **(B)** Display of the leading components in the human gut virome for individuals of enterotype 1 and 2.

Dissecting the human gut viral community’s component would offer a more complete picture. The numbers of vOTUs specific to three enterotypes were 198, 209, and 25 (Supplementary Table 7). They served as indicators to discover structures of human gut virome. We defined four components by classifying viruses in the human viral community into four categories: enterotype 1, 2, and 3 specific viruses, and the others (Figure 5A), called comp1, comp2, comp3, and comp4. The average relative abundance across samples in enterotype 1 were 0.26, 0.19, 0.01, and 0.54, respectively (Supplementary Table 10). The ones in enterotype 2 were 0.16, 0.31, 0.01, and 0.52. The comp1 and comp2 dominated in enterotype 1 and 2, respectively. This finding is expected, since enterotypes 1 and 2 contained very little comp3, while enterotype 3 had more comp3 component. Violin plots were shown to compare the relative abundance of comp1 and comp2 in different projects (Figure 5B). All of the projects reported significantly higher relative abundance of comp1 than comp2 across samples in enterotype 1. In contrast, the opposite trend was observed enterotype 2. This phenomenon indicated that comp1 and comp2 determined the viral enterotype assignment. The division of viral enterotypes showed more about relative abundance rather than absence of viral group.

The viral enterotype may play a dominant role in influencing the structural variance of the gut virome via a variety of factors. The Adonis test was used to determine the significance of viral enterotypes. The results were significant in all projects. Viral enterotypes explained the most structural variances in the gut virome (Figure 4D and Supplementary Table 11). In the Peru and cirrhosis projects, the Adonis R squared values were 0.62 and 0.57, respectively. The differences in groups by age, disease, BMI, raw data, and sex were not significant in all projects. BMI was second only to enterotype in explaining the most structural variance in the remaining factors (Supplementary Table 11). A probable explanation is the correlation between BMI and bile acid metabolism (Ma et al., 2014). Characterizing the interaction between the gut virome and external stimuli was complex. Whether a single factor has a particular contribution requires consideration of the context of this factor. An example of this situation is that, in liver cirrhosis, the association between the gut virome and age was strong, but it was not significant for diabetes.

**FIGURE 6:**
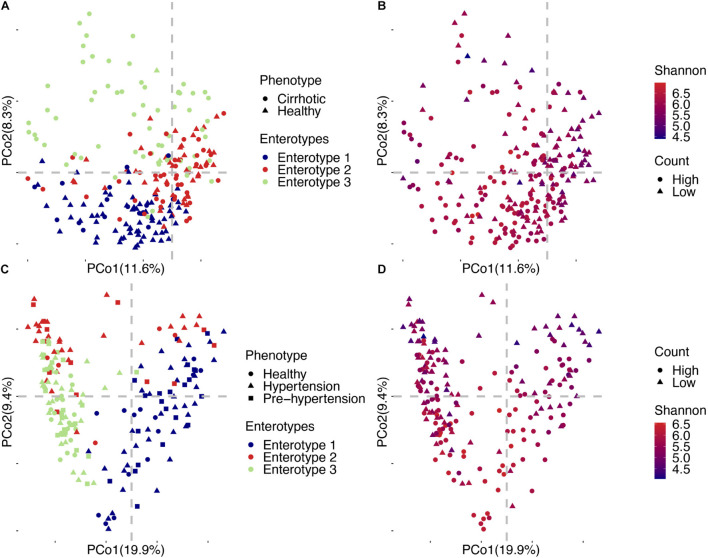
Detailed PCoA map of liver cirrhosis and hypertension. **(A)** Samples of liver cirrhosis were plotted in relation to their phenotype and enterotypes. **(B)** Samples of liver cirrhosis were plotted in relation to their viral count and Shannon index. **(C)** Samples of hypertension were plotted in relation to their phenotype and enterotype. **(D)** Samples of hypertension were plotted in relation to their viral count and Shannon index.

Enterotypes are useful for describing the gut microbial community, and determining the association between diseases and enterotype is important to detect high risk individuals in population. In the liver cirrhosis project, individuals could be broadly divided into three categories (Figure 6A). Enterotypes 1 and 3 were enriched in healthy individuals and patients, respectively (69 controls/16 cases vs. 2 controls/64 cases, Supplementary Table 8), and enterotype 2 accounted for half of them (43 controls, 43 cases, Supplementary Table 8). We found that the viral enterotype was significantly related to liver cirrhosis (Fisher’s exact test, *p* = 5.99E-24, Supplementary Table 12). Enterotype 3 was loosely distributed in individuals (Figure 5A). However, enterotypes 1 and 2 showed a closer relationship. These three groups did not have discrete clustering boundaries and demonstrated some overlap with one another in the PCoA plot. There was no apparent clustering of samples enriched locally due to the viral count or the Shannon index (Figure 6B). In the hypertension project, the clustering boundaries of enterotypes 1 and 3 were more pronounced than those for enterotype 2 (Figure 6C), and there was no overlapping area between the two clusters. This finding was surprising because individuals in enterotype 2 had a smaller viral count and a lower Shannon index (Figure 6D). Some of them were close to enterotype 1, while others had clusters of enterotype 3. However, the specific vOTUs and enrichment direction of individuals in enterotype 2 showed a high consistency (Figure 4B), indicating that enterotype 2 was real. We found no significant association between the viral enterotype and hypertension (Fisher’s exact test, *p* = 0.3, Supplementary Table 12). Gut virome community disorders showed significant differences in the cirrhosis and hypertension projects, which indicated that not all diseases caused evident ecological perturbation in the human gut. Thus, applying viral enterotypes as biomarkers for predicting clinical disease requires specific consideration.

## Discussion

Bacterial enterotypes have been deeply investigated in many studies on the human gut microbiome (Arumugam et al., 2011). They explored the human gut microbial compositions generally proposed into two enterotypes, dominated by *Bacteroidetes* and *Prevotella* and associated with digesting meat and vegetarian food (Arumugam et al., 2011; Costea et al., 2017). We examined the possibility to define the enterotypes by human gut virome and their relationship with human diseases. Our experiments observed that most individuals could be classified into two viral enterotypes, suggesting the viruses (most are phages) may consistently target particular bacteria. We found these two viral enterotypes were widespread in individuals by examining the vOTU enrichment trend (section “Materials and Methods,” Figure 4 and Table 2). Recent studies showed two common commensal dsDNA virus branches, crAssphage (Dutilh et al., 2014) and Gubaphage (Camarillo-Guerrero et al., 2021), might represent the observed two viral enterotypes in the human intestine. But it requires extensive analysis to make this conclusion. Zuo et al. (2020) also revealed two dominant enterotypes for viral communities from diverse ethnicities. Therefore, current evidence and our findings allow us to verify the existence of two discrete viral enterotypes (Figures 4, 5 and Table 2). Virus analysis revealed greater richness and Shannon index in enterotype 1 than enterotype 2 individuals (Figure 4). Enterotype 1 might have more stable viral ecological communities than enterotype 2. We found enterotype 2 had 1.38 times more patients than the ones in enterotype 1. This result suggests that a stable microbial community is a higher resilience response to unexpected external perturbations (Lozupone et al., 2012).

Besides the two enterotypes mentioned above, we observed another enterotype (enterotype 3) existed in several datasets. In the hypertension dataset (Li et al., 2017), enterotype 3 was not associated with the disease (Supplementary Table 12). We observed about half of vOTUs in enterotype 3, which co-appeared in enterotype 1. The remaining half vOTUs were shared with enterotype 2. It suggests the enterotypes 1 and 2 could be the two endpoints of the gradient distribution. Jeffery et al. (2012) observed a similar phenomenon in bacterial enterotypes. Unlike the hypertension project, enterotype 3 was associated with diseases in the liver cirrhosis project, as 64 of the 66 samples were from patients. We also observed the enterotype 3 was different across the studies in terms of vOTU enrichment (Figures 4, 5). This may be a potential mechanism behind enterotype 3 that was generated by the interaction between phenotypes and the gut virome. In the future study, we need to investigate more homogeneous “healthy” subjects to examine the distribution of viral enterotypes.

There was a lower similarity (larger BC distance, Figure 4C) across samples in the cirrhosis enterotype 3 cohort, but their virus counts were higher than enterotype 2. Given those parallels, evidence showed that bile acids are closely related to the human gut microbiome (Wahlström et al., 2016; Jia et al., 2018). A possible reason is that the disordered bile acid metabolism fundamentally altered the intestinal microenvironment in patients. It provided the opportunity to allow some viruses to colonize the human gut. This could be proved by dramatic viruses increases in the rarefaction curve (Figure 2). It may lead to a large-scale viral replacement and reduce similarities of virus in the patients (Shivanna et al., 2014; Hu et al., 2019). In contrast, we found a lower similarity in samples from the Sweden mother-child pair enterotype 3 cohort, with the viral count being significantly lower than the global average level. Young children’s digestive tract might enrich a vast collection of low-frequency viruses, but it also depends on the composition of their gut microbes (Derrien et al., 2019).

We examined the enterotypes on a China diabetes project based on bacterial and viral levels (Wang et al., 2012), and found there was a strong correlation between them (China T2D discovery: *p* = 1.70E-07; China T2D validation: *p* = 1.58E-11, Fisher’s exact test, Supplementary Table 13). We revealed both bacterial and viral enterotypes were not randomly distributed and that the bacteria had a strong selection on the viruses. They were not significantly correlated with sex, age, BMI, and disease (Supplementary Table 14). This finding may be explained by the limited use of high-abundance bacteria and viruses to assign enterotypes. Suppose the high abundance of bacteria or viruses is associated with the disease. In that case, it implies most people will be in disease states (Huang et al., 2020), which conflicts with the fact that most people are healthy, which explains why normal bacterial or viral enterotypes do not correlate with disease.

Although we found a strong correlation between bacterial and viral enterotypes, they were not supposed to be equivalent. Wang et al. (2012) used enterotype as a covariate to stratify human gut microbiomes in MWAS. It showed effective improvement in the power of hypothesis testing. We suggest considering bacterial and viral enterotypes as independent covariates in MWAS. We used MaAsLin2 to identify viruses that were specific to enterotypes and diseases. The number of disease-associated vOTUs was significantly lower than when considered the viral enterotype as a covariate in the cirrhosis dataset. There were 56 vOTUs identified to be associated with liver cirrhosis (*q*-value ≤ 0.05), if the enterotype was excluded. In contrast, we found 241 and 7 vOTUs were associated with enterotype and disease, respectively. There were 21 vOTUs identified as disease-related that became enterotype-associated. These results may hint viral enterotypes need to be taken into account in MWAS. Although many studies suggest that most phages are not strongly associated with disease, we cannot rule out the contribution of phages to the disease (Ma et al., 2018).

To better understand the contribution of enterotypes to diseases, we need an enterotype normalization method. Costea et al. (2017) previously reported one normalized pipeline for enterotypes. Researchers need to establish an enterotype database of the gut virome community based on a large health cohort. And build a machine learning enterotyping model and train it using this database. Finally, this model was used to predict the potential enterotypes of given samples on the basis of their viral abundance matrices. The heterogeneity among the data did not prevent us from discovering homogenous enterotypes in different projects. It demonstrated the ability to imply viral enterotypes comparison in various datasets. The construction of a large-scale viral enterotype database to define the enterotyping mathematical space of healthy individuals might be helpful to detect individuals with disease outside the mathematical space. Therefore, we believe that using viral enterotypes of the gut virome community as a feature for disease prediction will significantly improve the accuracy of disease prediction.

## Data Availability Statement

Publicly available datasets were analyzed in this study. This data can be found here: https://www.ncbi.nlm.nih.gov/sra. ERP002469; ERP004605; ERP005860; ERP005989; ERP006678; ERP008729; ERP010700; ERP012177; ERP015450; ERP016813; SRP008047; SRP011011; SRP045211; SRP052307; SRP080787; SRP090628; SRP100446; and SRP100575.

## Author Contributions

XF conceived this study. LS, LZ, and XF analyzed the data, prepared the figures, and drafted the manuscript. All authors contributed to the article and approved the submitted version.

## Conflict of Interest

The authors declare that the research was conducted in the absence of any commercial or financial relationships that could be construed as a potential conflict of interest.

## Publisher’s Note

All claims expressed in this article are solely those of the authors and do not necessarily represent those of their affiliated organizations, or those of the publisher, the editors and the reviewers. Any product that may be evaluated in this article, or claim that may be made by its manufacturer, is not guaranteed or endorsed by the publisher.

## Footnotes

1
https://www.ncbi.nlm.nih.gov/genome/viruses/

2
https://huttenhower.sph.harvard.edu/kneaddata/

3
https://cran.r-project.org/web/packages/metap/index.html

4
huttenhower.sph.harvard.edu/maaslin2
