# Supplementary material for: Characterizing Enterotypes in Human Metagenomics: A Viral Perspective
Source: Front Microbiol. 2021 Sep 29;12:740990. doi: 10.3389/fmicb.2021.740990 (PMC8511818; doi:10.3389/fmicb.2021.740990)
Supplement: Supplementary file 1 [file Presentation_1.PDF]

Supplementary Material

1 Supplementary Tables

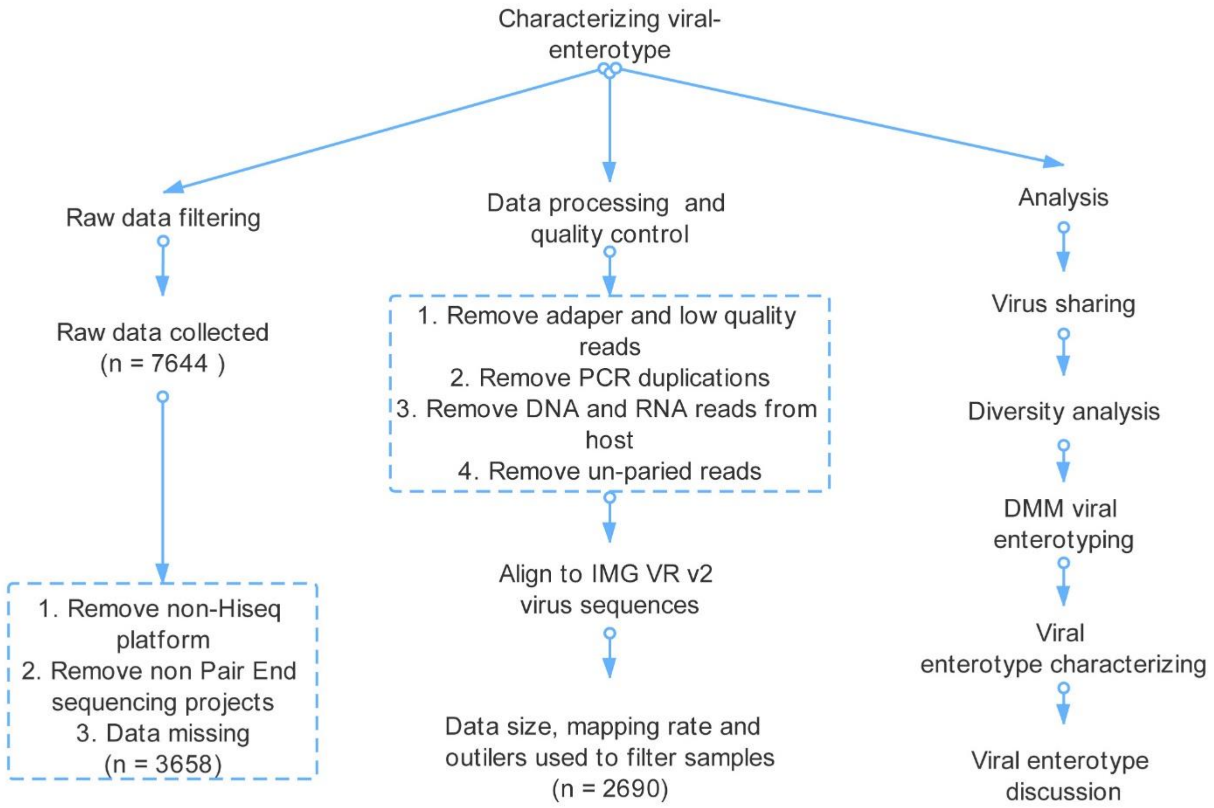

Supplementary Figure 1: Flowchart of the analysis pipeline.

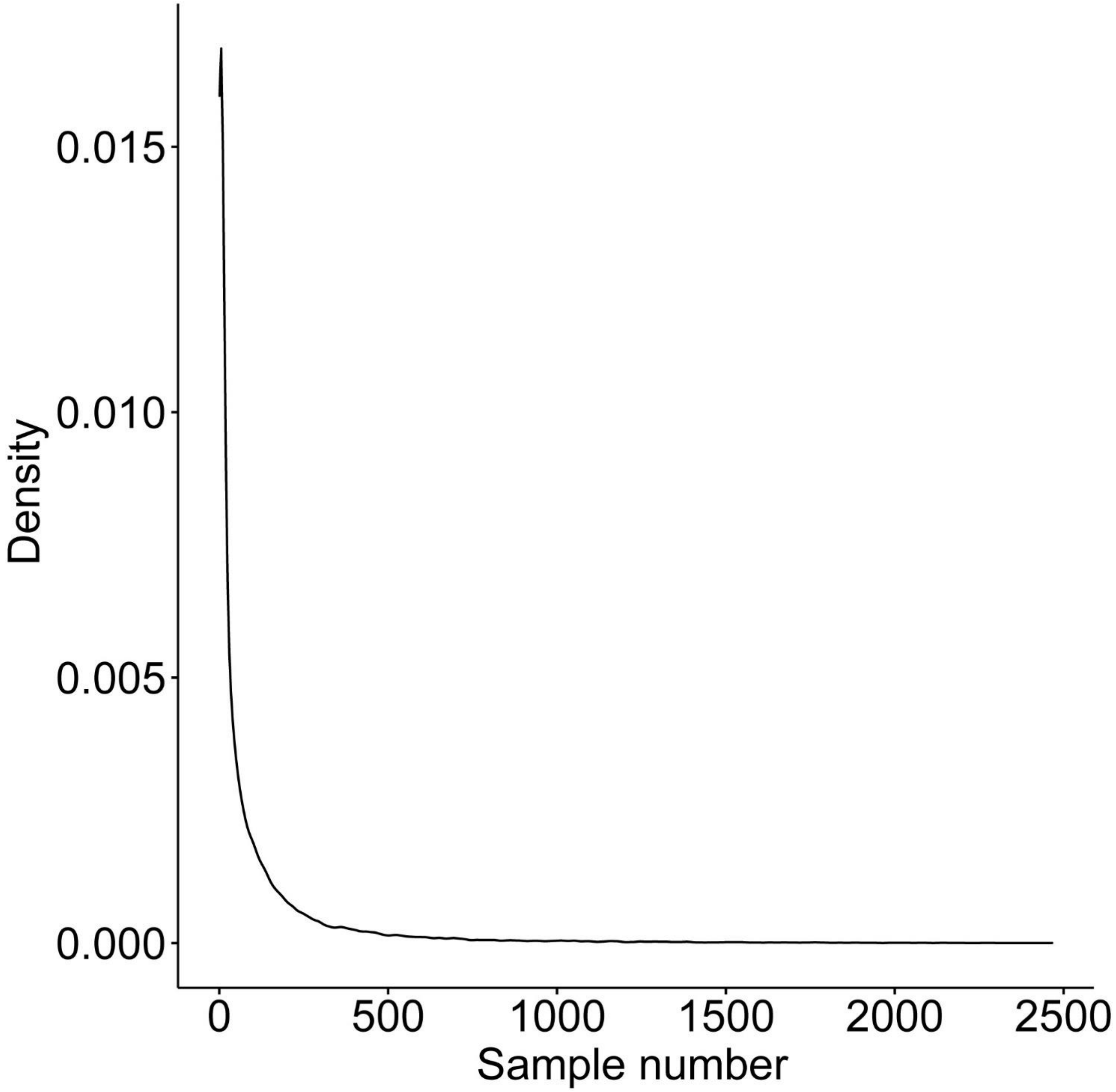

Supplementary Figure 2: Density distribution of sample numbers, x-axis indicates the total number of samples containing a particular virus.

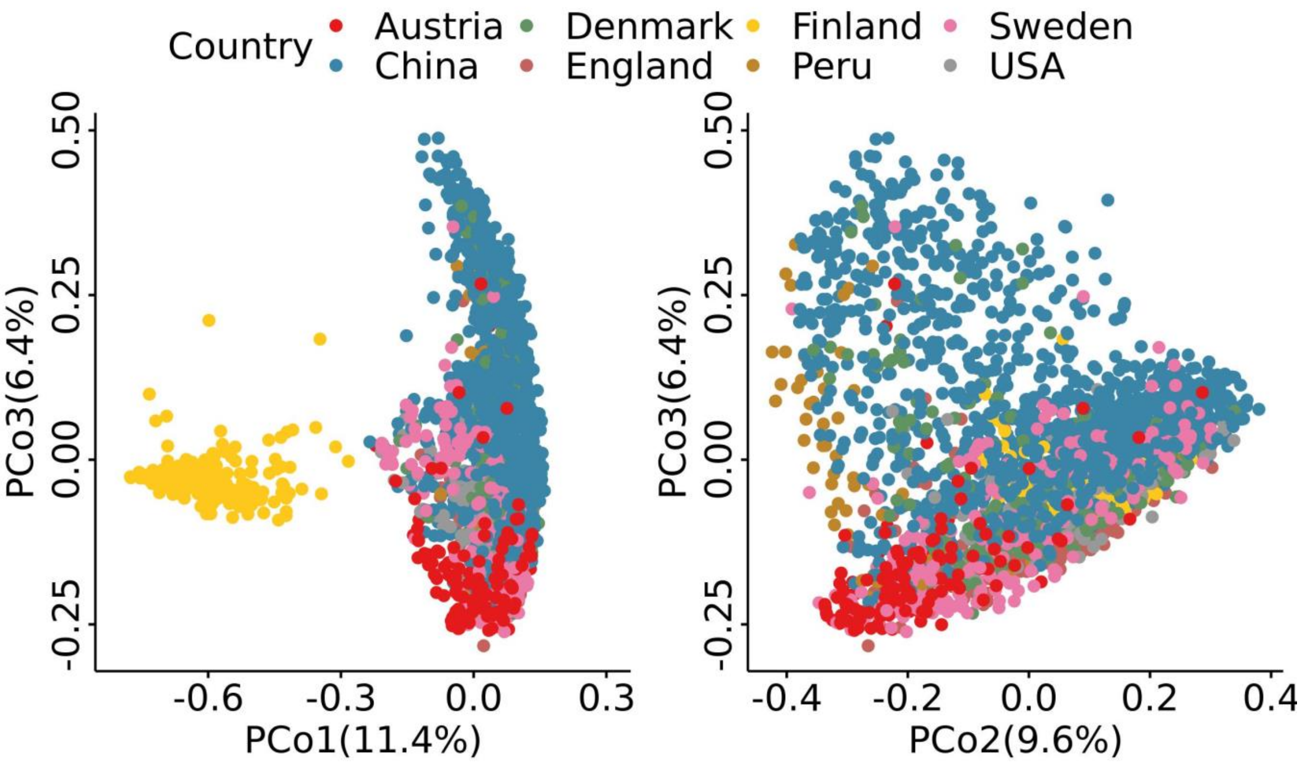

Supplementary Figure 3: PCoA based on the Bray–Curtis distance and the relative abundance of viruses.

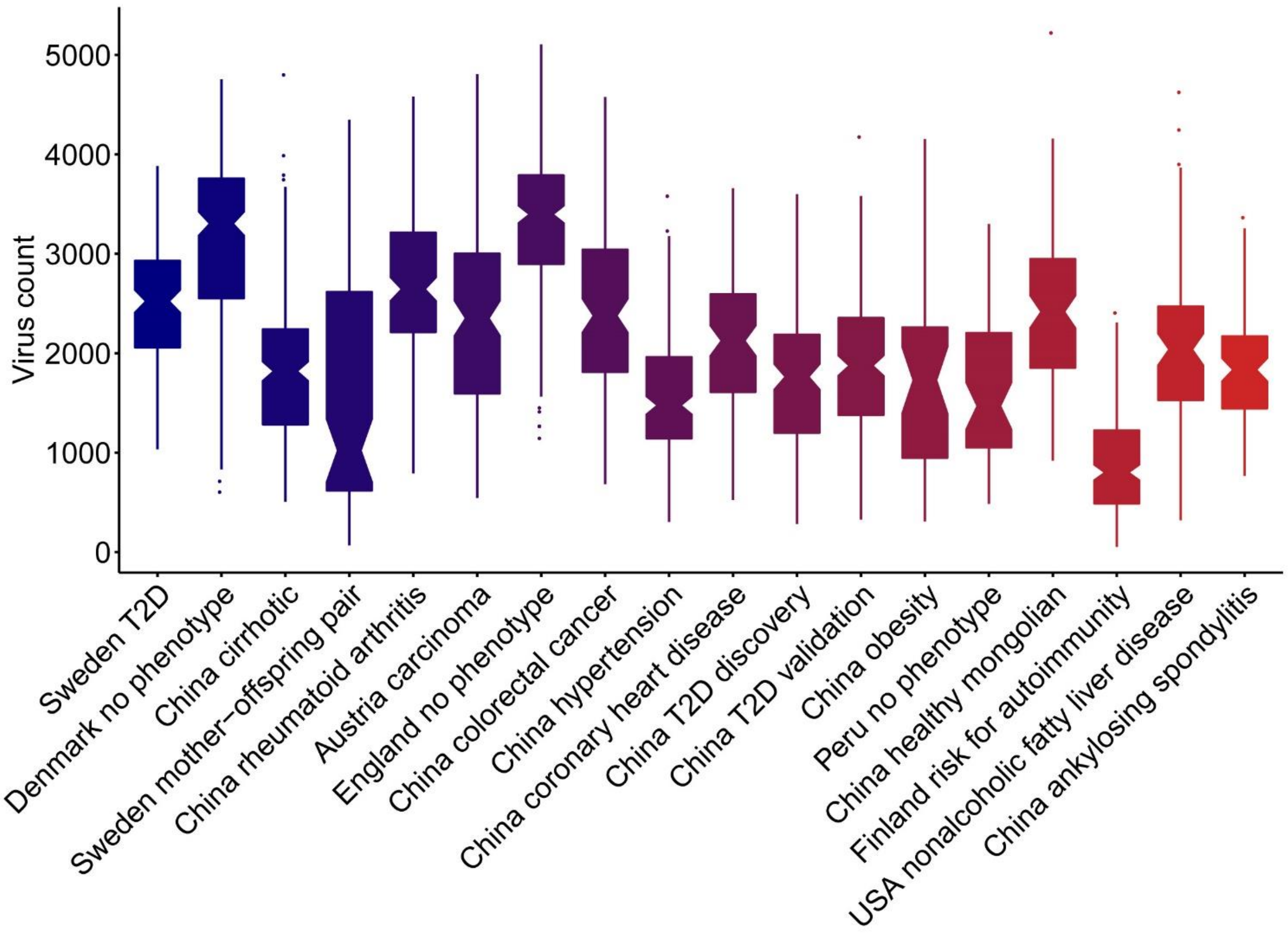

Supplementary Figure 4: The boxplot of virus count shows the number of virus detection in each project.

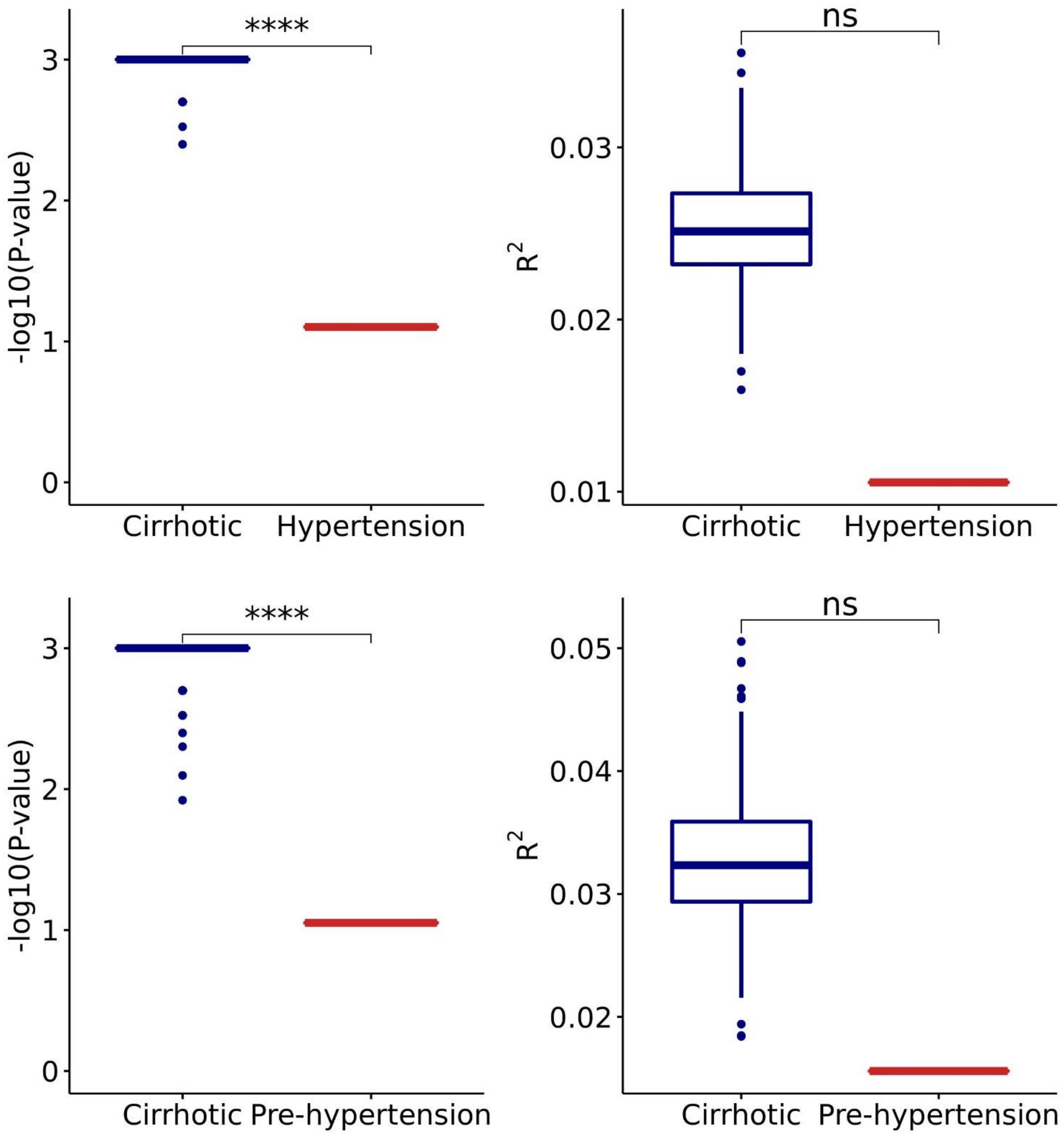

Supplementary Figure 5: PERMANOVA test for differences in control/case gut viral community in cirrhosis and hypertension data. We did 1000 permutations by randomly sampling the same number of individuals from two projects.

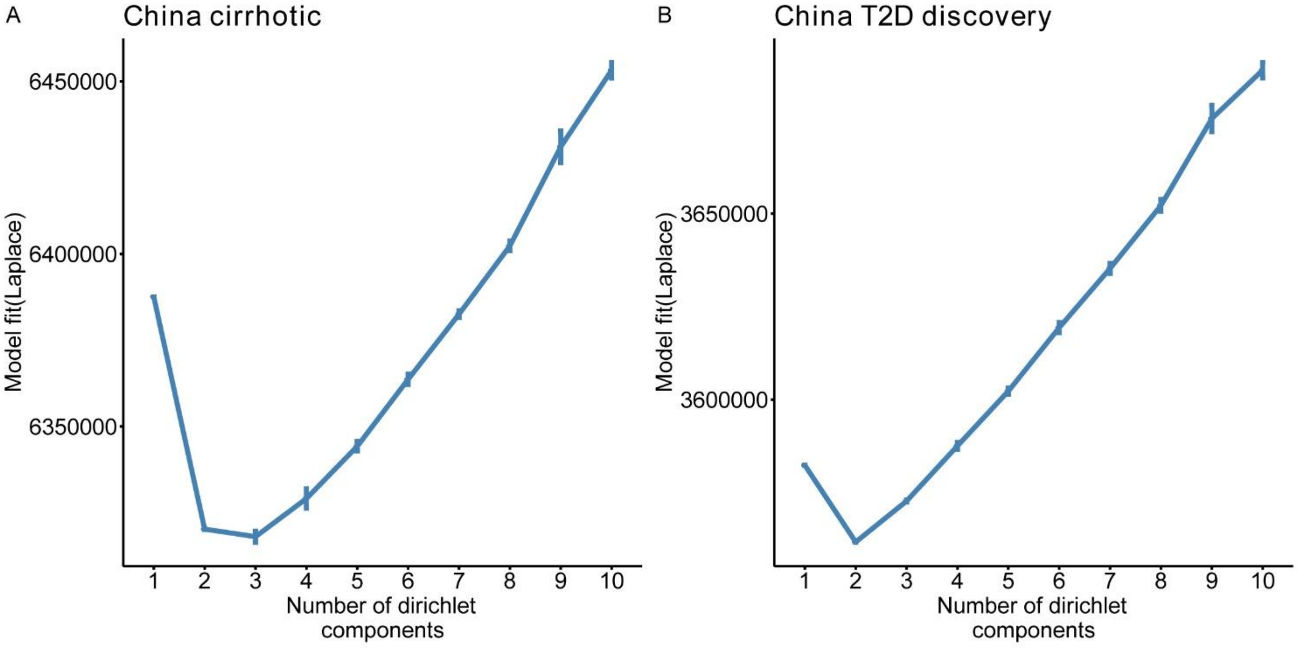

Supplementary Figure 6: Minimum Laplace determined enterotype assignment optimal results.

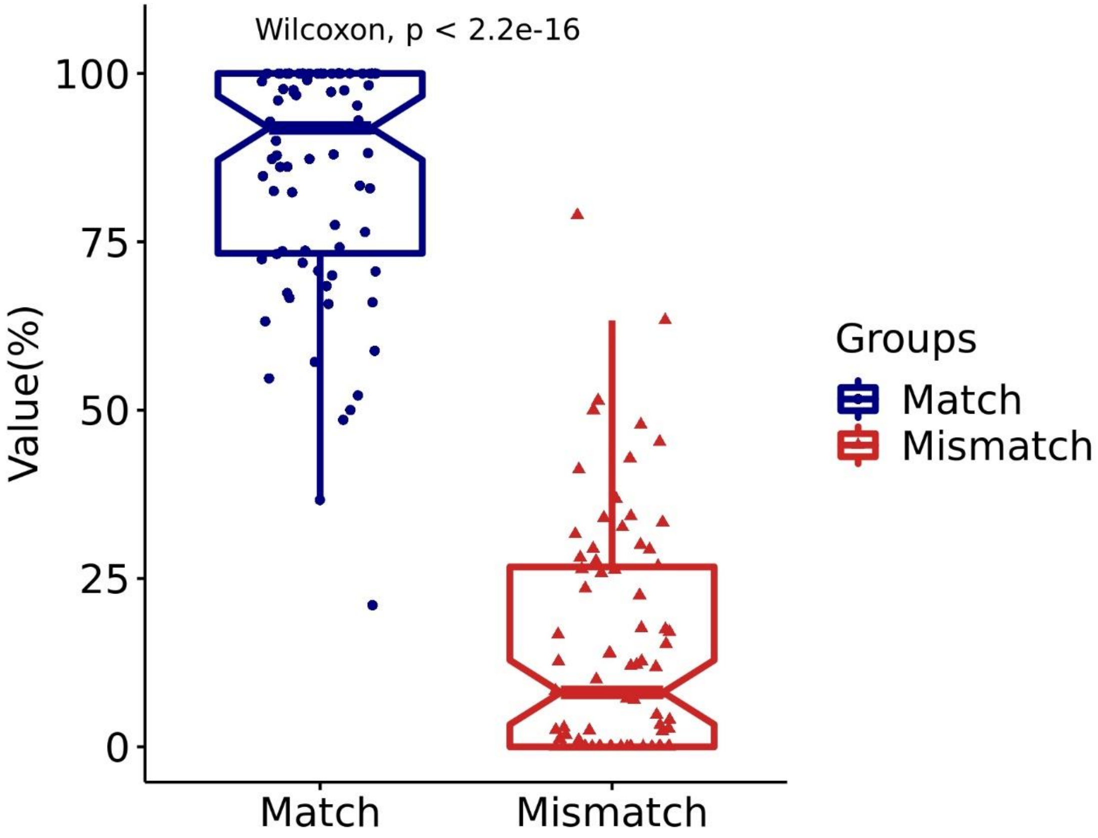

Supplementary Figure 7: We summarized the shared percentage of vOTUs specific to enterotype shared by the manually categorized groups in different projects, and compared to the mismatch ratio.
